# What are the effects of herbivore diversity on tundra ecosystems? A systematic review protocol

**DOI:** 10.1186/s13750-022-00257-z

**Published:** 2022-01-31

**Authors:** Isabel C. Barrio, Laura Barbero-Palacios, Elina Kaarlejärvi, James D. M. Speed, Starri Heiðmarsson, David S. Hik, Eeva M. Soininen

**Affiliations:** 1 Faculty of Environmental and Forest Sciences, Agricultural University of Iceland, Árleyni 22, Keldnaholt, 112 Reykjavík, Iceland; 2 Organismal and Evolutionary Research Programme, University of Helsinki, FI-00014 Helsinki, Finland; 3 Department of Natural History, NTNU University Museum, Norwegian University of Science and Technology, 7491 Trondheim, Norway; 4 Icelandic Institute of Natural History, Borgum við Norðurslóð, 600 Akureyri, Iceland; 5 Department of Biological Sciences, Simon Fraser University, Burnaby, BC V5A 1S6 Canada; 6 Department of Arctic and Marine Biology, UiT – the Arctic University of Norway, 9037 Tromsö, Norway

**Keywords:** Herbivore assemblage, Browsing, Grazing, Defoliation, Ecosystem function, Plant–herbivore interaction, Species richness

## Abstract

**Background:**

Changes in the diversity of herbivore communities can strongly influence the functioning of northern ecosystems. Different herbivores have different impacts on ecosystems because of differences in their diets, behaviour and energy requirements. The combined effects of different herbivores can in some cases compensate each other but lead to stronger directional changes elsewhere. However, the diversity of herbivore assemblages has until recently been a largely overlooked dimension of plant–herbivore interactions. Given the ongoing environmental changes in tundra ecosystems, with increased influx of boreal species and changes in the distribution and abundance of arctic herbivores, a better understanding of the consequences of changes in the diversity of herbivore assemblages is needed. This protocol presents the methodology that will be used in a systematic review on the effects of herbivore diversity on different processes, functions and properties of tundra ecosystems.

**Methods:**

This systematic review builds on an earlier systematic map on herbivory studies in the Arctic that identified a relatively large number of studies assessing the effects of multiple herbivores. The systematic review will include primary field studies retrieved from databases, search engines and specialist websites, that compare responses of tundra ecosystems to different levels of herbivore diversity, including both vertebrate and invertebrate herbivores. We will use species richness of herbivores or the richness of functional groups of herbivores as a measure of the diversity of the herbivore assemblages. Studies will be screened in three stages: title, abstract and full text, and inclusion will follow clearly identified eligibility criteria, based on their target population, exposure, comparator and study design. The review will cover terrestrial Arctic ecosystems including the forest-tundra ecotone. Potential outcomes will include multiple processes, functions and properties of tundra ecosystems related to primary productivity, nutrient cycling, accumulation and dynamics of nutrient pools, as well as the impacts of herbivores on other organisms. Studies will be critically appraised for validity, and where studies report similar outcomes, meta-analysis will be performed.

**Supplementary Information:**

The online version contains supplementary material available at 10.1186/s13750-022-00257-z.

## Background

**Fig. 1:**
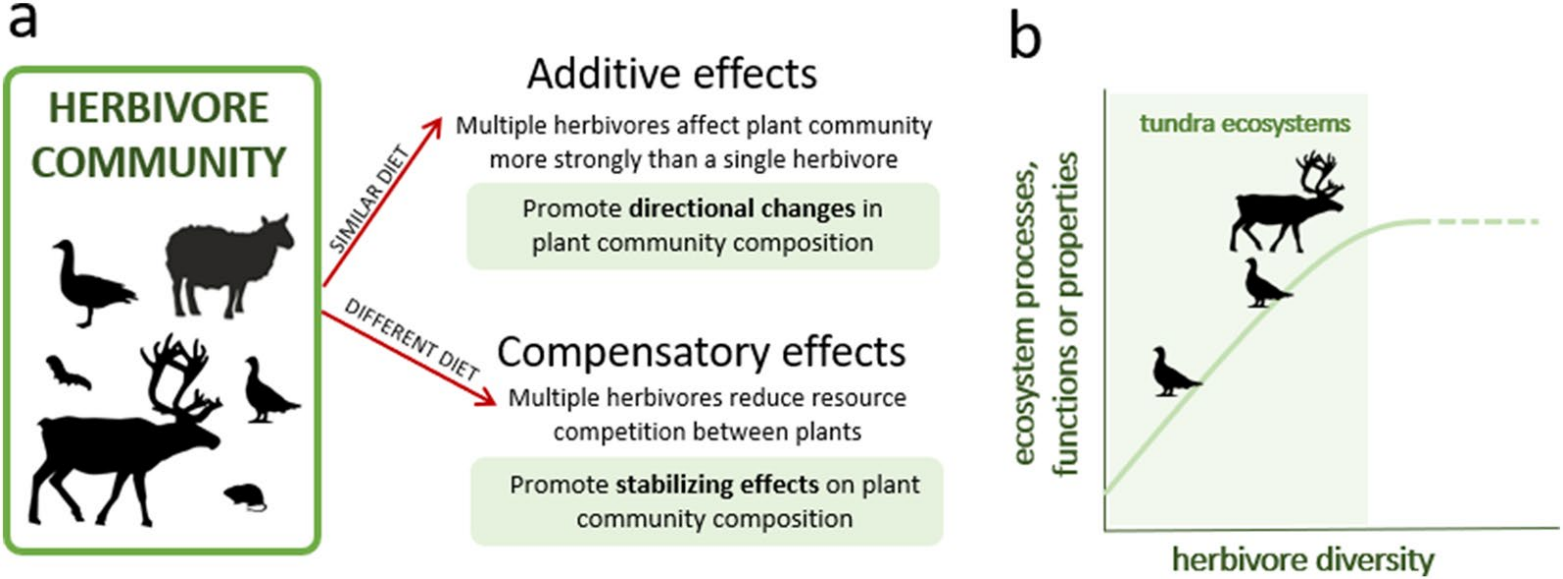
The diversity of the herbivore community (here expressed as richness of species or groups of herbivores) can have different impacts on processes, functions or properties of tundra ecosystems. **a** Taking as an example the effects of herbivores on vegetation, if the herbivores present in the herbivore community have similar diet we can expect additive effects on vegetation, where the combined effects of herbivores promote directional changes in plant community composition. On the other hand, if herbivores have different diets, we can expect compensatory effects, where the effects of different herbivores on plant species may balance each other by reducing competition among plant species and lead to stabilizing effects on plant community composition. **b** We hypothesize that herbivore diversity will influence ecosystem processes, functions or properties directly (e.g. consuming biomass, trampling) and indirectly (via effects on plant community composition as in panel **a**). Through both direct and indirect effects, we expect the combined effects of multiple herbivores on processes, functions and properties to be greater compared to the effects of single herbivores, although the slope of this relationship can change depending on the ecosystem process, function or property and the herbivore assemblage being considered. At very high herbivore diversity this relationship may saturate (dashed line) but we expect that in tundra ecosystems (shaded area), with relatively low numbers of species of herbivores, the relationship will not saturate

Herbivores in tundra ecosystems include organisms varying in size, from large ungulates, like muskoxen or reindeer, to small rodents, birds and invertebrates [1]. Arctic herbivores also vary in other traits that influence how they interact with plants and their environment, such as migratory behaviour, home range size, or their digestive physiology [2]. Given these differences, the diet choices and energy requirements of herbivores differ strongly [3, 4] and so do their impacts on ecosystems. Further, the effects of herbivores on processes, functions and properties of tundra ecosystems can be direct or indirect. For example, trampling by large herbivores can influence soil structure and soil biota directly, but also indirectly through changes in plant abundance and community composition [5]. To add to this complexity, the combined effects of different herbivore assemblages can lead to opposite outcomes (Fig. 1a). For example, in some cases the combined effects of large and small mammalian herbivores on vegetation are stronger than would be predicted for each group of herbivores alone [6–8]. In other cases, the effects of different herbivores can compensate each other if herbivores consume different plant species, leading to little to no changes in plant community composition [3, 9]. However, herbivore diversity has until recently been a largely overlooked dimension of plant–herbivore interactions in tundra ecosystems [10, 11], and we know little about how functionally different herbivore assemblages will affect these systems.

Tundra ecosystems are highly sensitive to environmental changes [12], and trophic dynamics are already responding to climatic changes [13, 14]. It has been suggested that the extent of ecosystem change in higher latitude systems at the end of the Pleistocene was determined by the loss of key species of herbivores [15], shifting in many cases vegetation from a grass-dominated steppe to a moss-dominated tundra [16]. Contemporary herbivore communities in tundra ecosystems are species-poor, compared to those present in the Pleistocene [17], but they can still drive ecosystem shifts [18, 19]. It has been hypothesized that a more diverse herbivore assemblage would have higher potential to influence ecosystem dynamics and drive transitions between vegetation states [17], so we are crucially in need of a better understanding of the status of herbivore assemblages and their role in tundra ecosystems. Knowledge on how the effects of different herbivores combine is thus particularly relevant to guide conservation and sustainable grazing management in tundra ecosystems that are grazed by wild and domestic herbivores [20].

We will use a systematic review to assess the effects of herbivore diversity on the functioning of tundra ecosystems. We will focus on multiple ecosystem processes, functions and properties related to primary productivity, nutrient cycling and nutrient pools, as well as the impacts of herbivores on other organisms, to comprehensively assess the role of herbivore diversity in the functioning of tundra ecosystems [21, 22]. By synthesizing the results of studies that evaluate the effects of different herbivore assemblages, we will be able to assess the influence of herbivore diversity on ecosystem functioning. As a measure of diversity, we will use the richness of species or groups of herbivores. We hypothesize that changes in the processes, functions or properties of tundra ecosystems will be affected by herbivore diversity (Fig. 1b). Specifically, we expect that increased diversity of herbivores will enhance ecosystem functioning through the complementarity of different herbivores, particularly in tundra ecosystems because they host relatively low herbivore diversity [2].

The topic for this systematic review was identified following the work on a systematic map [23] authored by a large number of scientists working on Arctic herbivory. These scientists represent the main stakeholder group for the topic of the present systematic review. Many of these scientists also work for conservation and environmental management agencies, thus broadening the scope to include other relevant stakeholders for the topic of the systematic review. Similar to the process followed by Soininen et al*.* [23], we will issue an open call for collaboration through the Herbivory Network (https://herbivory.lbhi.is/), to ensure openness and engagement of key participants in the systematic review.

## Objective of the review

The main objective of the systematic review is to synthesize available evidence on the effects of herbivore diversity on tundra ecosystems. A recent systematic map on the effects of herbivory on Arctic vegetation [23] identified a substantial number of studies (98) that investigated the effect of several herbivores on vegetation responses, indicating that a systematic review on this topic is possible. In the present review, we will extend the effects of herbivores to include non-vegetation functions, processes and properties in tundra ecosystems. Thus, we expect the number of studies to be larger than those identified by the systematic map, although this number will in turn be limited by the studies from which we can extract data and by our definition of the comparator (see components of the primary question and eligibility criteria below).

*Primary question.* What are the effects of herbivore diversity on processes, functions and properties of tundra ecosystems?

Components of the primary question

- *Population* terrestrial Arctic ecosystems
- *Exposure* herbivory (including disturbance and fertilization effects of herbivores)
- *Comparator* contrasting levels of herbivore diversity (species richness or richness of functional groups of herbivores)
- *Outcome* measured ecological processes, functions and properties in response to herbivory

## Methods

### Searching for articles

The search string for this systematic review will be the same as in a previously published systematic map [23, 24]. That search string was broad enough to include all potentially relevant studies for the present systematic review because it focused on the region/system (terrestrial Arctic ecosystems) and exposure (herbivory), which are common to the systematic map and the present systematic review. Yet, the search string did not pose any restriction on the outcome or comparator. In contrast to the systematic map, where the outcomes included the responses of plants to herbivory and the comparator was no herbivory or alternative levels of herbivory [24], the present systematic review focuses on the responses of ecological processes, functions and properties to herbivory, where the comparator is different levels of herbivore diversity. The search for the systematic map was conducted in February 2019 and retrieved 3200 records, which were filtered based on the eligibility criteria defined for the systematic map. Since the outcomes of interest of this systematic review are broader and include also other ecosystem components and a different comparator, we defined new eligibility criteria for the systematic review (see eligibility criteria below).

The search string comprises two substrings, one targeted at delimiting the study region and the other targeted at the exposure element (see details in [22]). Our full search string (formatted for Web of Science) is:

(arctic OR subarctic OR tundra) AND (herbivor* OR graz* OR browser OR browsing OR grubb* OR trampl* OR defolia* OR ((invertebrate OR insect) AND (gall* OR mining OR miner)))

The search will be conducted in English in global search sources, and English together with relevant local languages (Russian, French, Finnish, Swedish, Norwegian, Icelandic, and Danish) in searches from local/regional sources (Additional file 1).

Publications will be searched in the following global search sources:

- Scopus (article title, abstract and keyword search) with no further limitations applied.
- Web of Science (topic search), including all databases: Web of Science Core Collection, KCI-Korean Journal database, MEDLINE, Russian Science Citation Index and SciELO Citation Index.
- Google Scholar: title search standardized so that search history is not taken into account. We will only include the first 300 search results as recommended by Haddaway et al. [25].

Details for institutional subscriptions for the final searches will be reported. In addition, we will search in local and specialist databases for grey literature using the list of sources from [23] (Additional file 1). We will complement the bibliographic database searches by checking the reference lists in relevant articles to identify other potentially relevant articles (i.e. “snowballing” [26]). We will apply no time or document type restrictions for the search, but we will exclude publications for which we cannot access the full text, either in electronic or paper form.

#### Assessing the specificity and sensitivity of the search

The specificity (minimizing the proportion of irrelevant studies returned by the search) and sensitivity (finding all relevant studies) of the search string for the systematic review was assessed in scoping exercises during protocol development [24]. Further, the comprehensiveness of the search was tested against an independent test-list of articles that the protocol development team identified as relevant to answer the question of the systematic review (Additional file 2). In May 2021, searches using the search string returned 2135 hits in the Scopus database, 3037 within Web of Science and ‘about 4650’ hits on Google Scholar. All of the articles included in the test-list (20 articles) were included in the results from this search. We screened the titles of the records retrieved from Scopus to check the specificity of the search string. Following the same procedure as in Soininen et al*.* [24] we subsampled the first 500 records sorted alphabetically by first author name. Based on their titles, we excluded 52% of the records, mainly because they were not focusing on terrestrial ecosystems (71% of excluded documents). This result is similar to the systematic map (46% of documents were excluded; [24]), where the specificity of the search string was deemed adequate, as no additional search terms would screen away studies conducted in non-terrestrial ecosystems.

We assessed the sensitivity of the search string by comparing the records retrieved in Scopus by the full search string (combining the two substrings: region- and exposure-specific) to the substrings separately. A total of 115,964 records were identified by the region-specific search string that were not included in the full search string, while 226,264 records identified by the exposure-specific search string were not retrieved by the full search string. From each of these, the first 1000 records ordered alphabetically by first author name were screened for relevance, based on title, abstract and full text, to identify potentially relevant records that could have been missed by the combined search string. None of these records were deemed as potentially relevant.

### Article screening and study eligibility criteria

#### Screening process

Studies will be evaluated for inclusion in three stages: title, abstract and full text screening, based on the eligibility criteria presented below. When uncertain about whether a study should pass to the next stage of screening, reviewers will be inclusive. Reviewers will not take part in the critical appraisal of studies they have authored or co-authored. A list of studies excluded at full text stage with reasons for exclusion will be provided as an additional file to the systematic review.

**Fig. 2:**
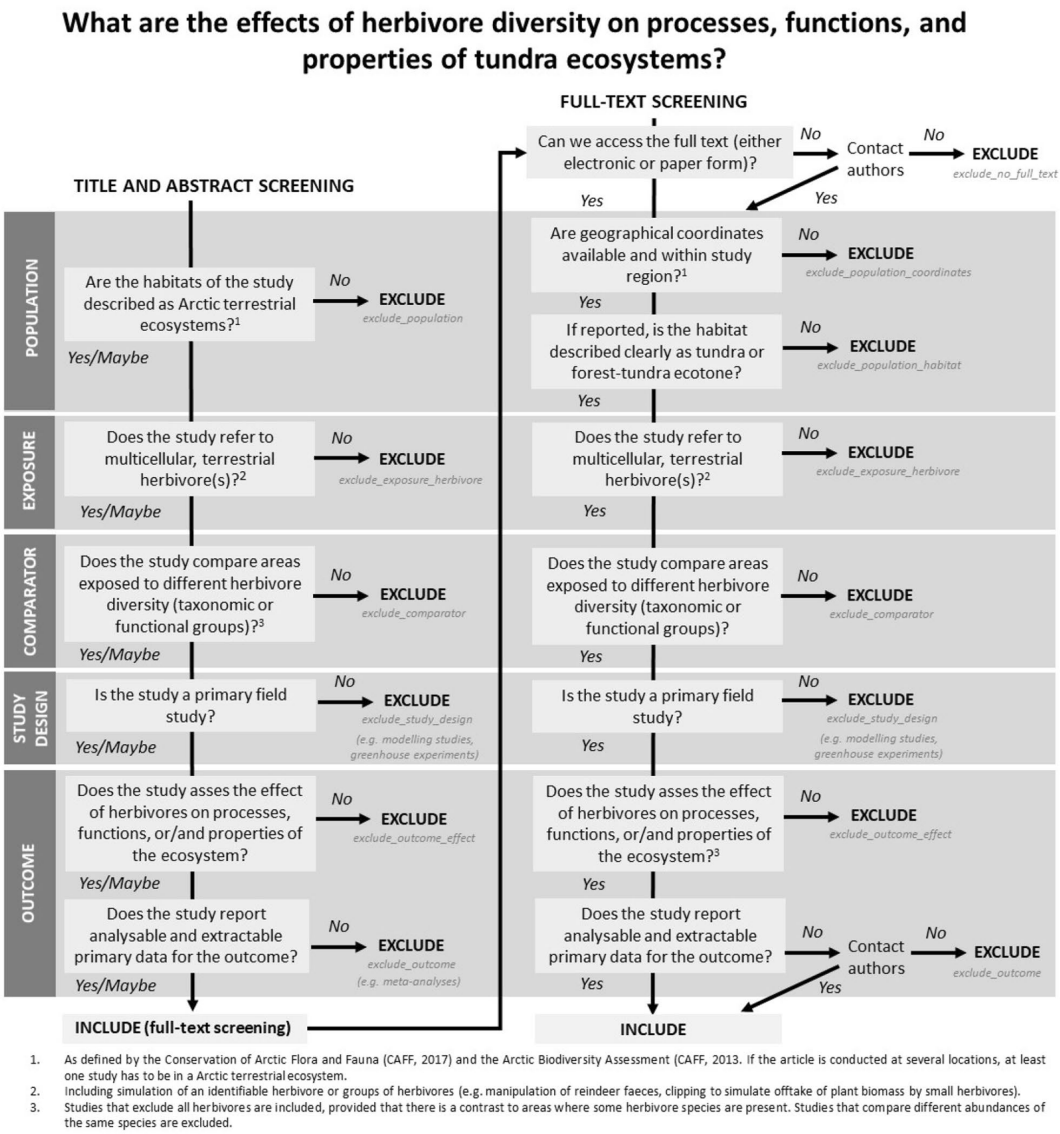
Decision tree for the title, abstract and full text screening stages

During the development of the systematic review protocol, we tested the repeatability of the screening process at the abstract stage. Four co-authors (EMS, JDMS, LBP, ICB) screened the abstracts of 100 publications (from a list of alphabetically ordered records previously assessed as relevant by one of the authors based on their title). The observers unequivocally agreed on the classification of 58 studies, either for inclusion (15) or exclusion (43). From the remaining 42 studies, 32 corresponded to cases where three observers agreed and one disagreed, while 10 cases corresponded to cases where two observers suggested inclusion and two suggested exclusion. Of those 42 studies, 13 focused on a single herbivore species and it was not clear whether areas without herbivores were included in the study (i.e. no eligible comparator); 7 studies referred to simulated herbivory, 7 were paleo-ecological studies where the data on herbivores was unclear. In 6 cases observers disagreed on whether the studies assessed effects of herbivores, 2 studies focused on effects on the herbivore itself, three potentially referred to non-Arctic systems (e.g. using locality names that were identified by some reviewers as non-Arctic but not by others) and 4 had study-specific issues. These disagreements were discussed, and the eligibility criteria were refined. Based on these discussions and following the recommendations of Foo et al. [27] we built a decision tree to guide the screening process at the title and abstract stage and at the full text stage (Fig. 2). Two observers (ICB, LBP) screened 100 additional abstracts using this decision tree and their agreement in including or excluding documents increased from 82 to 93%.

In the systematic review, we will include an assessment of the repeatability of the screening process, where 10% of the articles at each stage (title, abstract and full text) will be assessed by two reviewers. Cohen’s Kappa (κ) statistic will be calculated to assess the consistency among reviewers [28]. After double screening 10% of the articles, reviewers will discuss and reconcile the disagreements and adjust the inclusion criteria accordingly. If deemed necessary, double screening of another subset of articles will be performed.

#### Eligibility criteria

Studies will be screened for eligibility based on their target population, exposure, comparator and outcome, as well as for the type of study design.

##### Eligible population (terrestrial Arctic ecosystems)

To be included, studies must focus on Arctic terrestrial ecosystems. As in Soininen et al. [23] we define the Arctic using the southern limit of the subarctic as defined by the Conservation of Arctic Flora and Fauna [29] to delimit the study region. To further limit the scope to tundra and ecotone forests, we will exclude studies in areas South of the subarctic zone, based on the definition used in the Arctic Biodiversity Assessment [30]. Studies conducted clearly in boreal forests or other non-Arctic terrestrial habitats will be excluded. We will extract geographical coordinates provided in the text, maps or place names, and we will exclude studies where it is not possible to extract this information. Articles reporting studies conducted at several locations including both Arctic and non-Arctic sites will be included, but only for the part conducted within the Arctic provided that it is possible to separate data from Arctic and non-Arctic sites; otherwise, the article will be excluded.

##### Eligible exposure (herbivory)

We will include studies that assess effects of terrestrial herbivores on tundra ecosystems (i.e., grazing, browsing, trampling and other types of disturbance, such as fertilizing, digging or grubbing). Studies have to report the identity of the herbivores, irrespective of taxonomic resolution; for example, studies may report the species of herbivore (e.g. *Rangifer tarandus*) or a broader group of herbivores (e.g. small mammalian herbivores). We will limit the scope to include only multicellular herbivores; we will thus exclude grazing by unicellular organisms. We will include studies simulating herbivory, as long as they clearly connect their experimental manipulation to the activities of an identifiable herbivore or group of herbivores; for example, we will include studies adding or removing reindeer faeces, studies clipping plant biomass simulating biomass consumption by lemmings and voles, or those mimicking grubbing disturbances by geese.

##### Eligible comparator (contrasting herbivore diversity)

As a measure of herbivore diversity, we will use the richness of herbivores in the herbivore assemblage, either as taxonomic units (species or subspecies) or broader groups of species (e.g. small mammals, geese). We acknowledge that richness is a coarse measure of diversity that only takes into account the presence or absence of species (or groups) and not their relative abundances. The inclusion of abundance data for herbivore populations would provide a more nuanced understanding of the role of herbivores in the functioning of ecosystems [31], but reliable information is limited to a few species and only for some regions [30]. To be included, studies must assess the effect of different herbivore assemblages by comparing areas exposed to different numbers of herbivore species or groups of species (or no herbivore species). We will thus include studies that exclude all herbivores, provided that there is a contrast to areas where some herbivore species are present. However, we will exclude studies where the change in the herbivore assemblage is due to changes in the relative abundance of some herbivore species or groups but not in its diversity; for example, we will exclude studies that assess the impacts of changes in abundance of lemmings and voles, unless there is a change in the number of species in the herbivore assemblage (e.g. populations of one species go locally extinct). We will place no restrictions on the type of comparison (e.g. experimental treatments, changes across spatial or temporal gradients).

##### Eligible outcome (changes in ecological processes, functions and properties of terrestrial Arctic ecosystems)

We will include studies that assess the effects of herbivory on processes, functions and properties of Arctic terrestrial ecosystems, including their effects on other organisms. We will place no restriction on the ecosystem processes, functions and properties reported, but we will exclude studies that only report aspects of the herbivore itself and not its impacts on other ecosystem components (e.g. population dynamics of the focal herbivore, habitat selection studies). We will also exclude studies that do not report an outcome and those that present no analysable primary data for the outcome. We will therefore exclude reviews and book chapters, unless they contain original, primary data. However, we will include, at title and abstract screening, any corrections and erratum to published papers, as these can include relevant information for our review results; such documents will be identified as partly redundant.

##### Eligible type of study design

We will include primary field studies (observational or manipulative) comparing ecosystem processes, functions and properties in areas and/or time periods with different levels of herbivore diversity. Remote sensing studies will be included but not modelling studies as these do not represent direct effects of herbivores. We will not include greenhouse experiments because they restrict access of natural herbivores, but we will include common garden experiments where herbivores have free access to the experimental areas.

### Redundancy

We will exclude studies that report data that are reported in another study. In such cases, we will include the study presenting the longest time series or the greatest number of replicates. For example, if a study presents a spatial or temporal subset of a larger dataset presented in another study, when possible, we will include the larger study. We will assess this first by checking the references cited in the methods section for potential overlap, and by checking studies that were conducted at the same location following Soininen et al. [23].

### Study validity assessment

We will assess the validity of studies fulfilling all the eligibility criteria described above. Studies will be categorised as having high, medium or low susceptibility to bias based on seven criteria proposed in the new prototype CEE critical appraisal tool [32]. These criteria include an assessment of the risk of bias due to the presence of confounding variables, post-intervention or exposure biases, misclassified comparison variables (in the case of observational studies) or performance biases (in the case of experimental studies), detection biases, and risks of outcome reporting or assessment biases. Analyses in the systematic review will be conducted with and without studies with high susceptibility of bias to assess their influence on synthesis results. Study validity will be critically appraised by the reviewers conducting the data coding process based on the seven criteria (Additional files 3 and 4). During protocol development we assessed the repeatability of the critical appraisal of study validity as part of the assessment of the data coding and extraction processes (see data coding strategy below) and refined the criteria for study validity assessment as needed. In the systematic review, a random subset of at least 10% of studies will be appraised by a second reviewer (same as for the data extraction process). Information about study validity assessment will be provided for all included studies as an additional file to the systematic review.

### Data coding and extraction strategy

For each eligible article, we will extract information on the variables described in Table 1. One article can contain several studies, when separate parts of the article differ in terms of outcome, environmental context or methodological approach. Information will be recorded separately for each study. If an article reports an outcome separately for different habitats, sites, etc. we will consider them as separate studies. If studies report repeated annual measurements, we will extract data from the last measurement. In case of studies reporting repeated measurements over the growing season, we will extract data from the peak of the growing season (i.e. late July-early August in Arctic terrestrial ecosystems). When a study reports several comparisons of herbivore diversity levels, for example in studies using size-selective exclosures, pairwise comparisons will be extracted as separate studies. We will conduct our analyses using species richness of herbivores when studies report sufficient taxonomic detail, but also grouping species into relevant functional groups of herbivores [2].

**Table 1:** List of variables to code from the studies, including key sources of heterogeneity known to influence the effects of herbivores on tundra ecosystems

Topic	Coding variable	Variable description	Source
Article ID	author_list	List of authors	P
	title	Title of the publication	P
	year	Year of publication	P
	journal	Journal or publishing house	P
	language	Language of the publication	P
	study_ID	Unique ID number for study	C
	searchable_pdf	Whether the document allows for automated text searches	P
Study location	country	Country or region name	P
	locality	Site name describing the locality, as specified in the study	P
	coordinate_source	Whether coordinates are provided in the text, based on maps, figures or place names	P
	coordinates_N, coordinates_E	Geographic coordinates in decimal degrees	P
	elevation	Elevation (m a.s.l.)	P
	elevation_DEM	Elevation (m a.s.l.) extracted from digital elevation model	D
Study type	study_design	Whether the study involves experimental manipulations, observational approaches or whether the study design is unclear	C
	diversity_contrast	Approach used to create or assess the difference in herbivore diversity, such as exclosures or spatial contrasts	C
	size_selective_exclosures	Does the study use different exclosures that prevent access of different sized herbivores?	C
	spatial_extent	Size of the study area	C
	spatial_resolution	Spatial scale at which the outcome is reported for each study	C
	temporal_resolution	Interval between measurements of the outcome	C
	sampling_frequency	Frequency of measurements	C
	year_start	Year when the study started	P
	year_end	Year when the study finished	P
Exposure and comparator	herbivore_data	How is the composition of the herbivore assemblages assessed?	C
	herbivore_ID_higher_diversity	Species (or group) list in the areas with higher herbivore diversity	P
	herbivore_ID_lower_diversity	Species (or group) list in the areas with lower herbivore diversity	P
Outcome	measured_response_variable	What was measured in areas exposed to different herbivore diversity?	P/C
	reported_units	Units in which the response variable is reported; "unitless" if there is no unit	P
	value_higher_diversity	Value of the outcome in the higher diversity area	P
	value_lower_diversity	Value of the outcome in the lower diversity area	P
	value_type	Does the value refer to the mean, median, etc	P
	variability_higher_diversity	Variability of the outcome in the higher diversity area	P
	variability_lower_diversity	Variability of the outcome in the lower diversity area	P
	variability_type	Type of variability measurement (SE, confidence intervals (CI), etc.)	P
	sample_size_higher_diversity	Number of observations in the higher diversity area	P
	sample_size_lower diversity	Number of observations in the lower diversity area	P
	effect_size	Value of the comparison between areas with different diversity	P
	effect_size_variability	Variability of the comparison	P
	effect_size_variability_type	Type of variability measurement (SE, confidence intervals (CI), etc.)	P
	effect_size_direction	Direction of the comparison, or unclear	C
	effect_size_sample_size	Number of observations for the comparison if reported	P
	effect_size_type	Type of comparison (e.g. model estimate)	C
	effect_size_comments	Any additional comments	C
	statistical_test	Type of statistical test reported (e.g. paired t-test)	P
	statistical_test_value	Value of the test statistic	P
	statistical_test_df	Degrees of freedom of the statistic	P
	statistical_test_p	p-value of the statistical test	P
	outcome_comments	Any additional comments	C
	more_data	Is there data available in the paper that is not extractable in its current form?	C
Study validity assessment	bias_risk_criterion1	Biases due to uncontrolled confounding variables that influence both the herbivore diversity levels and the response variable	C
	bias_risk_criterion2	Biases arising from systematic differences in the selection of areas into the study or analyses after treatment	C
	bias_risk_criterion3or4	Biases arising from misclassification or measurement of contrasts of herbivore diversity (observational studies) or treatments (experimental studies)	C
	bias_risk_criterion5	Biases arising from systematic differences in measurements of outcomes	C
	bias_risk_criterion6	Biases in reporting of study findings	C
	bias_risk_criterion7	Biases due to error in applied statistical methods	C
	bias_risk_comments	Please indicate any relevant comments; if there were confounding variables please report them here	C
Context	distance_to_treeline	Distance (km) to southern border of arctic subzone E	D
	distance_from_coast	Distance (km) to the coast	D
	bioclimatic_zone	Bioclimatic zone A to E, or "other"	D
	temperature	Temperature related variable(s) extracted from WorldClim	D
	precipitation	Precipitation related variable(s) extracted from Worldclim	D
	growing_season	Duration of growing season (days)	D
	productivity	Value of NDVI (vegetation greenness)	D
	recent_warming	Extent of recent warming	D
	recent_greening	Extent of recent greening	D
	extent_of_recent__change	Extent of recent change in growing season length	D
	soil_chemistry	Soil chemistry	C
	soil_texture	Broad categories of soil texture	C
	soil_moisture	Soil moisture as described in the study	C
	soil_type	Soil type as reported by the authors	P
	soil_type_D	Soil type	D
	permafrost	Presence of permafrost	C
	permafrost_D	Presence of permafrost	D
	habitat_type	Habitat types using the broad categories defined in CAVM	C
	habitat_type_D	Habitat types	D
	management_focus	Whether the study is framed within a management context	C
	conservation_focus	Whether the study is framed within a conservation context	C

The coding variables are grouped into broader topics. Source indicates where the data is extracted from: P for publication, D for digital spatial data layers and C classified by the reviewers based on information available in the publication. More detailed descriptions, examples of the variables and references for the digital spatial data (D) are provided in Additional file 3

To be included, studies will have to provide quantitative data on the outcome variables, although this information may take different forms, including estimates of means, measures of variation (standard deviation, standard error, confidence intervals) and sample size for the different levels of herbivore diversity, values for the comparison (effect size) or results of a statistical test for the comparison between different levels of herbivore diversity providing enough information to enable calculation of effect sizes. For example, measures of variability should be reported by the studies or be calculable from the information provided in the study [33]. When possible, we will convert these comparisons to raw values, so that we can compare absolute effects rather than relative changes. If the study provides more than one type of data (for example the study reports both the statistical test for the comparison and the mean and variance for the levels of herbivore diversity being compared), all data will be extracted. For the outcome variables we will extract data from the text, tables and graphs, using image analysis software (ImageJ; [34]) when needed. When raw data are provided, we will calculate summary statistics. Data on potential effect modifiers will also be extracted (see next section). In some cases, it may be useful to ask authors of relevant studies for access to unpublished primary data or to ask for confirmation of missing or unclear information. During the protocol development, we created an Excel sheet with drop-down menus and open fields to ensure consistency during the data coding and data extraction process. This coding template includes additional information per variable (definition, potential values) and examples that help clarify the decision-making process (Additional file 3).

During protocol development we tested our data coding and extraction strategy to evaluate whether it was possible to extract the proposed variables and whether we had excluded potentially relevant variables or categories presented in the studies. Three co-authors (ICB, LBP, EK) coded four studies each from among the studies that had passed the title, abstract and full text screening stages, representing different challenges in the data extraction process. Three of these studies were common to all observers to test for consistency in coding, and one was different to identify additional issues where the data coding strategy had to be refined. While most of the variables coded by all co-authors were consistent, we identified several minor issues and reviewed the coding template accordingly. During the systematic review data from 10% publications will be recorded by two reviewers to assess the repeatability of the data extraction process. The extracted data records will be made available as additional files.

### Potential effect modifiers and reasons for heterogeneity

Extraction of meta-data from the studies will include data regarding key sources of heterogeneity (Table 1). The list of potential variables was identified based on expert knowledge and discussions with relevant stakeholders in the protocol development team, and included variables known to influence the effects of herbivores on tundra ecosystems, such as the geographical location of the study (latitude and longitude, distance to treeline), climate, soil type, vegetation or habitat type, proximity to human activities or human management of the herbivores (e.g. hunting, herding) or the ecosystem (e.g. protected areas), the presence and identity of predators and the identity of the herbivores in the herbivore assemblage. These potential modifiers will be used in meta-analyses to account for differences between studies. In most cases, these variables will be extracted from existing data layers or coded in the data extraction process (Additional file 3) to ensure that the information on these variables is extracted in a consistent way across studies (see e.g. [23]).

### Data synthesis and presentation

In the systematic review, we will describe the review process and the evidence base, focusing on the different ecosystem processes, functions and properties of terrestrial ecosystems in the Arctic that have been measured in the literature. The outcomes of this systematic review are purposely open-ended and will provide a sense of the scope and volume of the research that has been conducted on herbivore diversity and tundra ecosystems. This will in itself be a valuable output of the systematic review and the overview of outcomes measured by herbivore diversity studies will be summarized using descriptive statistics, tables and figures.

A narrative synthesis of the data will describe the quality of the results and the findings of studies taking into account the study validity assessment and will provide an overview of the evidence. Where enough studies (at least 5) report similar outcomes, meta-analysis will be undertaken [33], including in the models the effect of potential modifiers when possible. We will include studies that report outcomes for contrasting levels of herbivore diversity. Data will thus be often presented as average values and measures of variance for two groups separately. In such cases, we will calculate as an effect size the standardized mean difference (e.g. Hedges’ g), as the difference between group means standardized by the pooled standard deviation. Alternatively, when the outcomes refer to rates of change between the different groups, we may use the response ratio as a measure of effect size [33]. Ultimately, the type of effect size calculated will depend on how the outcomes are reported in these studies. In all cases, effect sizes will be standardized and weighted based on inverse variance. We will take into account the potential for non-independence of effect sizes arising from articles reporting several studies by including article ID as a random factor in our meta-analyses. These mixed-effects models allow assessing whether the heterogeneity in effect sizes arises from the effects of moderator variables or random sources by using inverse variance weights adjusted for the estimated random effects component [33]. Where possible, publication bias and sensitivity analysis will also be conducted using diagnostic plots, like funnel plots of effect size vs study size and the trim-and-fill method [35].

We will present the results using the following illustrations:

- A flow diagram illustrating the inclusion/exclusion process including the number of papers retained at each stage of the process (ROSES diagram; [36]).
- A geographic map showing the locations of the studies included in the systematic review
- Scatterplots, plots of means or barplots showing the relationship between herbivore assemblages and different ecosystem processes, functions and properties (mean effect sizes and variance)
- Forest plots will summarize meta-analysis outputs, separating by groups where at least five studies are included in each group.

For effective communication of results we will publish the final dataset in full on an interactive map server, where readers can explore and filter the results and visualize results (see for example [23]). Finally, we will include as an additional file to the systematic review a check-list of reporting standards for systematic reviews (i.e. ROSES form), as we do for this systematic review protocol (Additional file 5).

## Supplementary Information

**Additional file 1.**List of local and specialist databases for grey literature searches.

**Additional file 2.** Test-list of articles used to assess the comprehensiveness of the search.

**Additional file 3.** Data coding template.

**Additional file 4.** Criteria for study validity assessment.

**Additional file 5.** ROSES form for systematic review protocol.

## Data Availability

All data generated or analysed during this study will be included in the published article and its supplementary information files.
