# Supplementary material for: What are the effects of herbivore diversity on tundra ecosystems? A systematic review protocol
Source: Environ Evid. 2022 Jan 31;11:1. doi: 10.1186/s13750-022-00257-z (PMC11378829; doi:10.1186/s13750-022-00257-z)
Supplement: Supplementary file 4 — Additional file 4. Criteria for study validity assessment. [file 13750_2022_257_MOESM4_ESM.pdf]

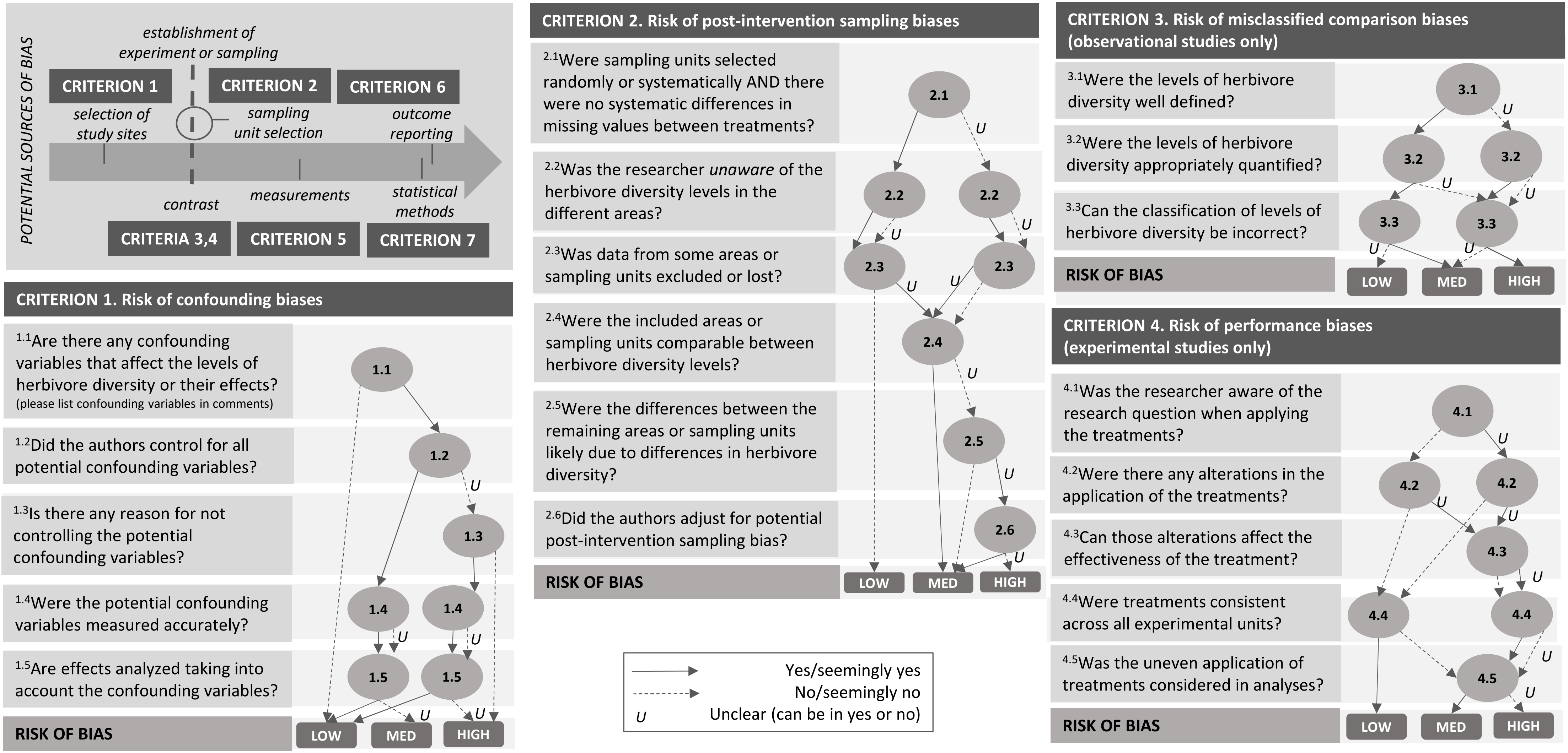
POTENTIAL SOURCES OF BIAS
establishment of experiment or sampling
CRITERION 1
selection of study sites
CRITERION 2
sampling unit selection
CRITERION 6
outcome reporting
contrast
measurements
statistical methods
CRITERIA 3,4
CRITERION 5
CRITERION 7
CRITERION 1. Risk of confounding biases
1.1 Are there any confounding variables that affect the levels of herbivore diversity or their effects? (please list confounding variables in comments)
1.2 Did the authors control for all potential confounding variables?
1.3 Is there any reason for not controlling the potential confounding variables?
1.4 Were the potential confounding variables measured accurately?
1.5 Are effects analyzed taking into account the confounding variables?
RISK OF BIAS
1.1
1.2
1.3
1.4
1.4
1.5
1.5
U
LOW
MED
HIGH
CRITERION 2. Risk of post-intervention sampling biases
2.1 Were sampling units selected randomly or systematically AND there were no systematic differences in missing values between treatments?
2.2 Was the researcher *unaware* of the herbivore diversity levels in the different areas?
2.3 Was data from some areas or sampling units excluded or lost?
2.4 Were the included areas or sampling units comparable between herbivore diversity levels?
2.5 Were the differences between the remaining areas or sampling units likely due to differences in herbivore diversity?
2.6 Did the authors adjust for potential post-intervention sampling bias?
RISK OF BIAS
2.1
2.2
2.2
2.3
2.3
2.4
2.5
2.6
U
LOW
MED
HIGH
CRITERION 3. Risk of misclassified comparison biases (observational studies only)
3.1 Were the levels of herbivore diversity well defined?
3.2 Were the levels of herbivore diversity appropriately quantified?
3.3 Can the classification of levels of herbivore diversity be incorrect?
RISK OF BIAS
3.1
3.2
3.2
3.3
3.3
U
LOW
MED
HIGH
CRITERION 4. Risk of performance biases (experimental studies only)
4.1 Was the researcher aware of the research question when applying the treatments?
4.2 Were there any alterations in the application of the treatments?
4.3 Can those alterations affect the effectiveness of the treatment?
4.4 Were treatments consistent across all experimental units?
4.5 Was the uneven application of treatments considered in analyses?
RISK OF BIAS
4.1
4.2
4.2
4.3
4.4
4.4
4.5
U
LOW
MED
HIGH
Yes/seemingly yes
No/seemingly no
U Unclear (can be in yes or no)

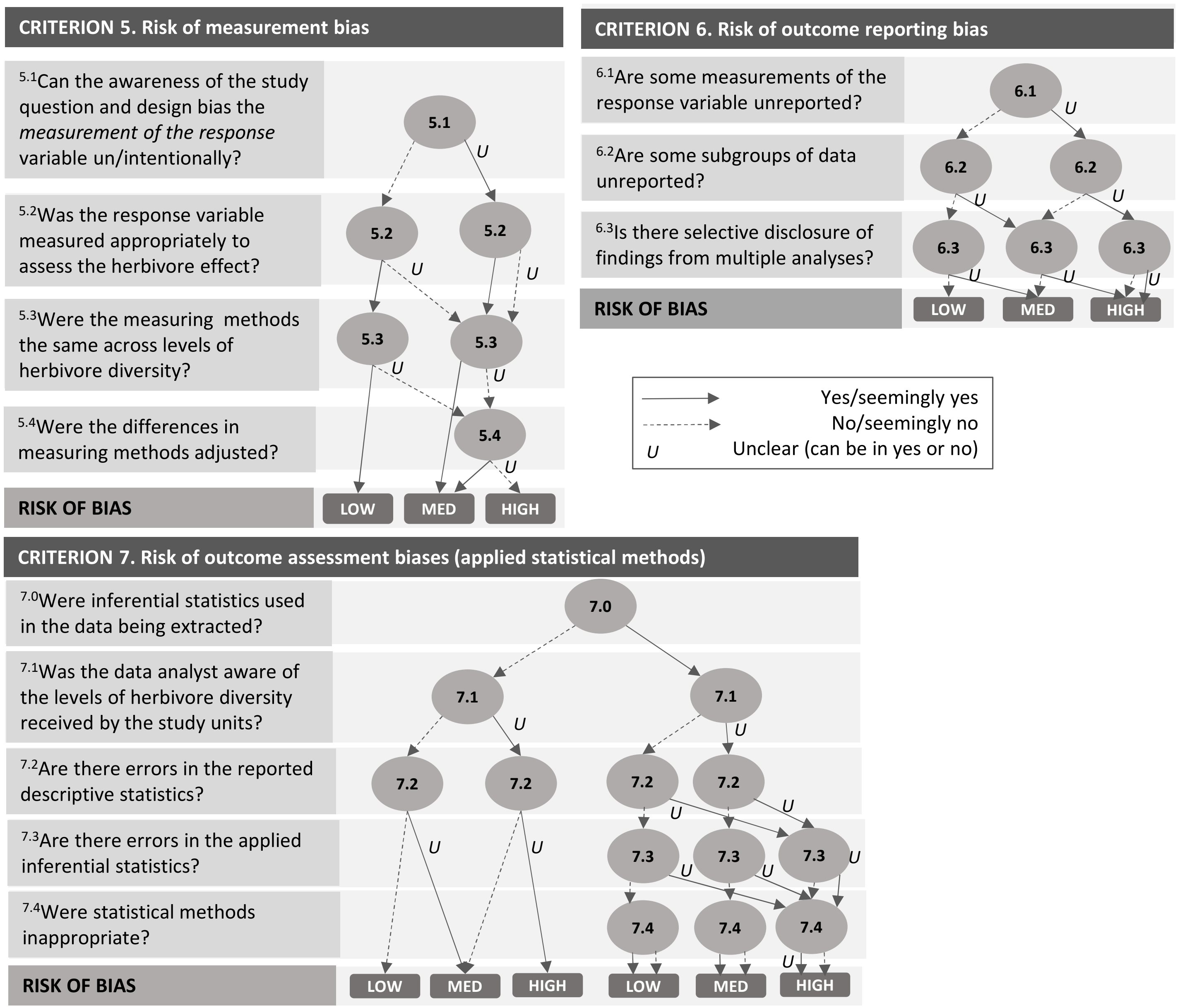
CRITERION 5. Risk of measurement bias
5.1 Can the awareness of the study question and design bias the measurement of the response variable un/intentionally?
5.2 Was the response variable measured appropriately to assess the herbivore effect?
5.3 Were the measuring methods the same across levels of herbivore diversity?
5.4 Were the differences in measuring methods adjusted?
RISK OF BIAS
5.1
5.2
5.2
5.3
5.3
5.4
U
LOW
MED
HIGH
CRITERION 6. Risk of outcome reporting bias
6.1 Are some measurements of the response variable unreported?
6.2 Are some subgroups of data unreported?
6.3 Is there selective disclosure of findings from multiple analyses?
RISK OF BIAS
6.1
6.2
6.2
6.3
6.3
6.3
U
LOW
MED
HIGH
Yes/seemingly yes
No/seemingly no
U Unclear (can be in yes or no)
CRITERION 7. Risk of outcome assessment biases (applied statistical methods)
7.0 Were inferential statistics used in the data being extracted?
7.1 Was the data analyst aware of the levels of herbivore diversity received by the study units?
7.2 Are there errors in the reported descriptive statistics?
7.3 Are there errors in the applied inferential statistics?
7.4 Were statistical methods inappropriate?
RISK OF BIAS
7.0
7.1
7.1
7.2
7.2
7.2
7.2
7.3
7.3
7.3
7.4
7.4
7.4
U
LOW
MED
HIGH
LOW
MED
HIGH
