# Supplementary material for: What are the effects of herbivore diversity on tundra ecosystems? A systematic review protocol
Source: Environ Evid. 2022 Jan 31;11:1. doi: 10.1186/s13750-022-00257-z (PMC11378829; doi:10.1186/s13750-022-00257-z)
Supplement: Supplementary file 5 — Additional file 5. ROSES form for systematic review protocol. [file 13750_2022_257_MOESM5_ESM.pdf]

| Section / sub-section | Topic | Description | Further explanation | Checklist/Meta-data | Author response | Comments |
|---|---|---|---|---|---|---|
| Title | Title | The title must indicate that it is a systematic review protocol, and must | The title should normally be the same or very similar to the review | Meta-data | What are the | the title indicates that the document |
| Type of review | Type of review | Select one of the following types of review: systematic review, systematic | See CEE Guidance on amendments and updates [1] | Meta-data | systematic | |
| Authors contacts | Authors contacts | The full names, institutional addresses, and email addresses for all authors | | Checklist | Yes | |
| Abstract | Structured summary | Abstract must not exceed 350 words and must include two sections 1) | | Checklist | Yes | |
| Background | Background | Describe the rationale for the review in the context of what is already | A theory of change and/or conceptual model can be presented that links | Checklist | Yes | |
| Stakeholder engagement | Stakeholder engagement | The planned/actual role of stakeholders throughout the review process | | Checklist | Yes | |
| Objective of the review | Objective | Describe the primary question and secondary questions (when applicable). | The primary question is the main question of the review. Secondary | Checklist | Yes | |
| | Definitions of the question | Break down and summarise question key elements e.g. population, | For other question types see [3,4] | Meta-data | Key elements of | |
| Methods | | | | | | |
| Searches | Search strategy | Detail the planned search strategy to be used, including: database names | Details regarding search strategy testing should be provided. | Checklist | Yes | |
| | Search string | Provide Boolean-style full search string and state the platform for which | | Meta-data | Full search string | |
| | Languages – bibliographic | List languages to be used in bibliographic database searches. | | Meta-data | English | |
| | Languages – grey literature | List languages to be used in organizational websites searches and web- | | Meta-data | English, Russian, French, Finnish, Swedish, Norwegian, Icelandic, and Danish | |
| | Bibliographic databases | Provide the number of bibliographic databases to be searched. | | Meta-data | 50 | list provided in Additional file 2 |
| | Web – based search engines | Provide the number of web – based search engines to be searched. | | Meta-data | 50 | list provided in Additional file 3 |
| | Organisational websites | Provide the number of organisational websites to be searched. | | Meta-data | 50 | list provided in Additional file 4 |
| | Estimating the comprehensiveness | Describe the process by which the comprehensiveness of the search | | Checklist | Yes | All articles included in a test-list (Additional file 2) were included in the search; sensitivity and specificity of the search string were also assessed during protocol development |
| | Search update | Describe any plans to update the searches during the conduct of the | Optional. A search update is good practice if original searches were | Checklist | n/a | The aim is to develop the systematic |
| Article screening and study | Screening strategy | Describe the methodology for screening articles/studies for | | Checklist | Yes | |
| | Consistency checking | Describe clearly the process for checking consistency of decisions including | | Checklist | Yes | Preliminary assessment of the |
| | Inclusion criteria | Describe the inclusion criteria used to assess relevance of identified | | Checklist | Yes | |
| | Reasons for exclusion | State that you will provide a list of articles excluded at full text with reasons | | Checklist | Yes | |
| Critical appraisal | Critical appraisal strategy | Describe here the method you propose for critical appraisal of study | | Checklist | Yes | |
| | Critical appraisal used in synthesis | Describe how the information from critical appraisal will be used in | | Checklist | Yes | |
| | Consistency checking | Describe how repeatability of critical appraisal of study validity will be | | Checklist | Yes | |
| Data extraction | Meta-data extraction and coding | Describe the method for meta-data extraction and coding for studies | | Checklist | Yes | |
| | Data extraction strategy | Describe the method for extraction of qualitative and/or quantitative study | | Checklist | Yes | |
| | Approaches to missing data | Describe any processes for obtaining and confirming missing or unclear | | Checklist | Yes | |
| | Consistency checking | Describe how repeatability of the meta-data/data extraction process will | | Checklist | Yes | Data will be extracted by a main |
| Potential effect modifiers/reasons | Potential effect modifiers/reasons | Provide a list of and justification for the effect modifiers /reasons for | The list should not be exhaustive but a short list of those variables thought | Checklist | Yes | Potential modifiers and sources of |
| Data synthesis and presentation | Type of synthesis | State the type of synthesis conducted as part of the systematic review | | Meta-data | narrative and | |
| | Narrative synthesis strategy | Describe methods to be used for narratively synthesising the evidence base | Vote-counting  (tallying of studies based on the direction or significance of | Checklist | Yes | |
| | Quantitative synthesis strategy | If data are appropriate for quantitative synthesis, describe planned | Compulsory if appropriate for data | Checklist | Yes | |
| | Qualitative synthesis strategy | Describe methods to be used for synthesising qualitative data and justify | Compulsory if appropriate for data | Checklist | Yes | |
| | Other synthesis strategies | Describe any other approaches to be used for synthesising data or | Compulsory if appropriate for data | Checklist | Yes | |
| | Assessment of risk of publication | Describe planned methods for examining the possible influence of | For quantitative syntheses this may be done using diagnostic plots or | Checklist | Yes | |
| | Knowledge gap identification | Describe the methods to be used to identify and/or prioritise key | Optional | Checklist | n/a | |
| | Demonstrating procedural | Describe the role of systematic reviewers (who have also authored articles | Reviewers who have authored articles to be considered within the review | Checklist | Yes | |
| Declarations | Competing interests | Describe of any financial or non-financial competing interests that the | | Checklist | Yes | |

**References**

[1] Bayliss, H.R., Haddaway, N.R., Eales, J., Frampton, G.K. and James, K.L., 2016. Updating and amending systematic reviews and systematic maps in environmental management. Environmental Evidence, 5(1), p.20.

[2] Haddaway, N.R., Kohl, C., da Silva, N.R., Schiemann, J., Spök, A., Stewart, R., Sweet, J.B. and Wilhelm, R., 2017. A framework for stakeholder engagement during systematic reviews and maps in environmental management. *Environmental Evidence* , *6* (1), p.11.

[3] Collaboration for Environmental Evidence. 2018. Guidelines and Standards for Evidence synthesis in Environmental Management. Version 5.0. www.environmentalevidence.org/information-for-authors.

[4] Leeds Institute of Health Sciences. https://medhealth.leeds.ac.uk/info/639/information_specialists/1500/search_concept_tools. Accessed 12/11/2017.
